# Bodily self and immune self: is there a link?

**DOI:** 10.3389/fnhum.2014.00138

**Published:** 2014-03-18

**Authors:** Marcello Costantini

**Affiliations:** ^1^Laboratory of Neuropsychology and Cognitive Neuroscience, Department of Neuroscience and Imaging, University G. d'AnnunzioChieti, Italy; ^2^Institute for Advanced Biomedical Technologies, University G. d'AnnunzioChieti, Italy; ^3^Mind, Brain Imaging and Neuroethics, Institute of Mental Health Research, University of OttawaOttawa, ON, Canada

**Keywords:** bodily self-consciousness, multisensory integration, immune system, immunity, sense of ownership

Normally we experience the physical body as our own, except in rare conditions and transitory illusions. Cognitive neuroscientists, philosophers, and psychologists call this feeling *bodily self-consciousness* (Damasio, 2003; Gallagher, 2005; Legrand, 2006; Blanke and Metzinger, 2009; Ferri et al., 2011, 2012). The Rubber Hand Illusion is though to operationalize on bodily self-consciousness (David et al., 2013, for a different view). By using this illusion it has been demostrated that bodily self-consciousness is highly variable across healthy individuals (Haans et al., 2012). This means that the susceptibility to the illusion largely varies across individuals. The origin of such variability remains largely unknown. In this opinion paper, I propose the possibility that it can be traced, in part, to the individual's immune system ability to differentiate the self from nonself. In particular, I suggest a biological substrate for such dependency.

The intriguing idea of a link between the Bodily Self and the Immune Self was proposed by Antonio Damasio more than 10 years ago (Damasio, 2003). Here the Bodily Self is defined as a non-propositional representation of intero-exteroceptive information (Haggard et al., 2003; Pacherie, 2008); and the Immune Self is defined as whatever the immune system recognize as belonging to the hosting body (Nossal, 2001). Damasio's fascinating suggestion remains largely at the theoretical level, with little empirical support. However, recently things are changing. Barnsley and colleagues found that during the Rubber Hand Illusion the immune system treats the concealed hand as a foreign body part, as demonstrated by increases histamine reactivity (Barnsley et al., 2011). Thus, Barnsley et al's data suggest a biological link between bodily self-consciousness and the immune system.

The immune system is a set of molecules, proteins and processes that protects against disease. To do that, the immune system must be able to distinguish the host's own cells from virus and bacteria. In principle the immune system has the potentiality to destroy the host (i.e., the Self) and the pathogens (i.e., the nonself). When the immune system appropriately regulates by engaging in biodestructive activity of a pathogen, then that pathogen is considered as part of the “nonself,” while tolerance towards the healthy tissue of the host is considered part of the “self.” However, when biodestructive activity is inappropriately regulated, autoimmunity might occur. Autoimmunity is indeed the error of the immune system in recognizing its own cells as nonself. Prominent examples include Celiac disease, diabetes mellitus type 1, systemic lupus erythematosus, Hashimoto's thyroiditis, Graves' disease, rheumatoid arthritis, and multiple sclerosis. The presence of an autoimmune disease is signaled by the appearance of autoantibodies in the patient blood. Among immunologists, different mechanisms have been proposed to explain the ability of the immune system to differentiate pathogens from the host, including altered self (Houghton, 1994), danger theory (Matzinger, 2002) and discontinuity theory (Pradeu et al., 2013). However, a detailed review of such proposals is far from the aim of the present paper.

How it is possible to explain an empirical link between the Bodily Self and the Immune Self? One possibility is to look at the basic mechanisms driving the Bodily Self and to look whether such mechanisms are sensitive to the status of the immune system.

According to some authors, the foundation of the self lies in the brain networks representing the body (Damasio, 2003; Gallagher, 2005; Blanke and Metzinger, 2009) and integrating interoceptive and exteroceptive inputs (Tsakiris et al., 2011). In the case of the rubber hand illusion, multisensory integration between vision and touch “changes the phenomenal status of the rubber hand from inanimate object to part of one's own body” (Ehrsson, 2012). Since multisensory bodily inputs are continuous, whereas external objects of perception are transient and therefore not continuous, these multisensory bodily inputs have been proposed as the basis for the Bodily Self (Arzy et al., 2006; Aspell et al., 2009; Ionta et al., 2011a,b, 2014; Limanowski and Blankenburg, 2013). According to this view, when multisensory bodily signals (i.e., visual and spatio-temporal signals) received from a body part match, a sense of ownership then arises for that body part (Ehrsson, 2012). Such constitutive role of multisensory integration in bodily self-consciousness might bridge the gap between the Bodily Self and the Immune Self. Indeed, despite its crucial role, multisensory integration is not an innate ability. Neurons with multisensory capability in the superior colliculus (SC) are ineffective in combining cross-modal stimuli at early stages of life (Wallace and Stein, 1997, 2001, 2007). They require postnatal maturation to acquire this capability.

Supporting this view is the finding that manipulating the spatio-temporal features of cross-modal stimuli has a strong impact on the normal development of multisensory neurons (Wallace et al., 2004). That is, changing the natural statistics of the environment (Chandrasekaran et al., 2009), changes the way neurons encode future stimuli (Wallace and Stein, 2007). This data support the idea that axon pruning and neural plasticity are necessary conditions for the maturation of multisensory neurons.

Strikingly, neural plasticity in the SC and other brain structures is deeply intertwined with the immune system via substances called cytokines, with the interleukins being prominent examples (Boulanger, 2009; Yirmiya and Goshen, 2011). These cells are used by the immune system to orchestrate the production of antibody and memory cell formation. The name interleukins mirrors their role in communication between leukocytes.

Interestingly, the prolonged exposure to pro-inflammatory cytokines causes premature stabilization of developing synapses within the tectum (i.e., the SC in mammals). Therefore, this prevents normal synaptic refinement and elimination that occurs during development. Indeed, during development, the optic tectum undergoes a substantial amount of modification, both locally (e.g., within the tectum itself) and in its projection to the retina (Lee et al., 2010). A considerable amount of this modification involves consolidation and stabilization of some synapses, and weakening and elimination of others. This evidence is in line with the protracted period of postnatal maturation required by multisensory neurons to develop the ability of combining cross-modal stimuli (Stein et al., 1973; Wallace and Stein, 1997, 2001). Other evidence of the link between neural plasticity and immune molecules comes from studies on long-term potentiation (LTP). In 1998, Schneider et al. demonstrated that the pro-inflammatory cytokine IL-1 in the hippocampus plays a pivotal role in long term potentiation as well as in maintaining it. The dose-dependent effect of IL-1 on memory and LTP is also demonstrated by the detrimental effect of intracerebroventricular administration of IL-1 on the acquisition of spatial memories after spatial water maze training (Oitzl et al., 1993). In the same vein, chronic exposure to IL-1 produces important deficit in spatial memory, as measured by means of the water maze paradigm, as well as diminished long-term contextual fear memory (Moore et al., 2009; Hein et al., 2010). Other cytokines, genes, and proteins play a role in the communication between the brain and the immune system (Boulanger and Shatz, 2004; Boulanger, 2009). For instance, several studies showed a negative effect of elevated IL-6 levels on synaptic plasticity (Li et al., 1997).

In summary, a basic sense of Self in housed in the brain structures representing the body (Legrand, 2006; Blanke, 2012). Such representations depend upon multisensory bodily signals. To develop the ability to integrate bodily signals axon pruning, neurogenesis and synaptic plasticity are mandatory processes (Stein et al., 1973). These processes, in turn, are deeply intertwined with the immune system (Boulanger, 2009; Yirmiya and Goshen, 2011). From this perspective, the Bodily Self and the Immune Self have a common biological background. I am not proposing a new theory of the Self, but I am proposing an extension to the theoretical framework that is being developed to better understand the Self. The immune system consists of many proteins, molecules and processes. Hence, my proposal is speculative and not exhaustive. Future empiric studies should be conducted to test this proposal.
